# The effects of a low carbohydrate diet combined with partial meal replacement on obese individuals

**DOI:** 10.1186/s12986-023-00740-5

**Published:** 2023-03-30

**Authors:** Yulian Zhong, Ximin Chen, Chao Huang, Yuexiao Chen, Fengyi Zhao, Runhua Hao, Niannian Wang, Wang Liao, Hui Xia, Ligang Yang, Shaokang Wang, Guiju Sun

**Affiliations:** 1 Key Laboratory of Environmental Medicine and Engineering of Ministry of Education, Department of Nutrition and Food Hygiene, School of Public Health, Southeast University, Nanjing, Jiangsu 210009 China; 2 Beijing Institute of Nutritional Resources, Beijing, 100069 China; 3 Institute of Biotechnology and Health, Beijing Academy of Science and Technology, Beijing, 100089 China

**Keywords:** Low carbohydrate diet, Obesity, Substitute meal, Weight-loss

## Abstract

**Objective:**

We explored the dietary effects of replacing normal dietary staple foods with supplementary nutritional protein powder, dietary fiber, and fish oil on several metabolic parameters. We examined weight loss, glucose and lipid metabolism, and intestinal flora in obese individuals when compared with individuals on a reduced staple food low carbohydrate diet.

**Methods:**

From inclusion and exclusion criteria, 99 participants (28 kg/m^2^ ≤ body mass index (BMI) ≤ 35 kg/m^2^) were recruited and randomly assigned to control and intervention 1 and 2 groups. Physical examinations and biochemical indices were performed/gathered before the intervention and at 4 and 13 weeks post intervention. After 13 weeks, feces was collected and 16s rDNA sequenced.

**Results:**

After 13 weeks, when compared with controls, body weight, BMI, waist circumference, hip circumference, systolic blood pressure, and diastolic blood pressure values in intervention group 1 were significantly reduced. In intervention group 2, body weight, BMI, waist circumference, and hip circumference were significantly reduced. Triglyceride (TG) levels in both intervention groups were significantly reduced. Fasting blood glucose, glycosylated hemoglobin, glycosylated albumin, total cholesterol, and apolipoprotein B levels in intervention group 1 were decreased, while high density lipoprotein cholesterol (HDL-c) decreased slightly. Glycosylated albumin, TG, and total cholesterol levels in intervention group 2 decreased, while HDL-c decreased slightly, High sensitive C-reactive protein, MPO, Ox-LDL, LEP, TGF-β_1_, IL-6, GPLD1, pro NT, GPC-4, and LPS levels in both intervention groups were lower when compared with controls. Adiponectin (ADPN) levels in intervention groups were higher when compared with controls. Tumor necrosis factor-α (TNF-α) levels in intervention group 1 were lower when compared with controls. There is no obvious difference in α diversity and β diversity between intestinal flora of 3 groups. Among the first 10 species of Phylum, only the control group and the intervention group 2 had significantly higher Patescibacteria than the intervention group 1. Among the first 10 species of Genus, only the number of Agathobacter in intervention group 2 was significantly higher than that in control group and intervention group 1.

**Conclusions:**

We showed that an LCD, where nutritional protein powder replaced some staple foods and dietary fiber and fish oil were simultaneously supplemented, significantly reduced weight and improved carbohydrate and lipid metabolism in obese individuals when compared with an LCD which reduced staple food intake.

**Supplementary Information:**

The online version contains supplementary material available at 10.1186/s12986-023-00740-5.

## Introduction

Obesity is a rapidly growing public health problem in both developed and developing countries [1]. Obesity increases the morbidity risks of hypertension, cardiovascular disease, diabetes, high cholesterol, cancer, respiratory disease, and musculoskeletal disease, with mortality risks gradually increasing once the overweight threshold is exceeded [2, 3].

A low carbohydrate diet (LCD) limits carbohydrates and replaces them with fat and/or protein. Both protein and fat cause satiety and reduce blood glucose fluctuations that lead to hunger. Molecularly, this occurs via insulin-mediated signal pathways that send appetite signals to the brain [4]. When compared with low-fat diets, individuals on LCD only need to reduce carbohydrate intake, fat and protein are consumed normally which improves LCD compliance,while those on low-fat diets limit calories [4].

Crowd compliance is critical for weight loss. One method improving compliance is to use partially controlled conventional foods or meal substitutes that provide predetermined food and calorie quantities. Partial diet control appears to improve dietary compliance by reducing participant tendencies to underestimate calorie intake [5, 6]. In particular, meal substitutes reduce the complexity associated with meal-planning and food preparation, reduce cognitive needs and decision-making, and reduce the implications of overeating [7]. Additionally, substitute meals support adherence to calorie goals via sensory-specific satiety [5].

Research now shows that increasing soybean protein intake reduces body fat rates, total serum cholesterol, and low-density lipoprotein cholesterol (LDL-c) levels [8]. Dietary fiber regulates blood lipids and sugars via physical effects, immune regulation, anti-inflammatory effects, and prebiotic effects so as to prevent and treat obesity. Fish oil combined with weight-reducing diets can significantly reduce waist circumference and waist hip ratios [9]. Cereals are rich in carbohydrates, with Chinese populations mainly eating rice and flour (wheat). Therefore, in obese individuals, we explored the advantages/disadvantages of replacing some staple foods with nutritional protein powders, dietary fiber, and fish oil when compared with an LCD where staple food intake was reduced. We hypothesized this scientific and healthy nutritional intervention diet could help obese individuals reduce weight.

## Methods

### Participants

#### Inclusion criteria

(1) Males or females aged between 18 and 65 years old with 28 kg/m^2^ ≤ BMI ≤ 35 kg/m^2^. (2) No serious liver, kidney, digestive tract, cardiovascular, cerebrovascular, or mental diseases. (3) No drug/dietary supplements lowering blood lipids, blood glucose, or weight levels within the previous 3 months: participants agreed to not use supplements/drugs during the intervention. (4) Dietary control/guidance acceptance by participants. (5) After information was provided, participants volunteered and signed consent forms.

#### Exclusion criteria

(1) Participants aged < 18 or > 65, BMI < 28 kg/m^2^ or BMI > 35 kg/m^2^. (2) Pregnant or lactating females. (3) Participants with serious diseases such as liver, kidney, digestive tract, cardiovascular, cerebrovascular, or psychosis. (4) In the previous 3 months, participants had taken drugs related to lowering blood lipids, blood glucose, or weight levels, and also dietary supplements potentially impacting study outcomes. (5) Signs of alcoholism. (6) Participants with special eating habits: vegetarians, ketogenic eaters. (7) Participants who could not follow study requirements.

### Groups and interventions

According to the formula N = 2 (Z_1−α/2_+Z_β_) 2σ^2^/d^2^ and the literature, each group had 28 individuals, which was based on a 10% follow-up rate loss. Using aforementioned criteria, 99 participants were recruited from Nanjing and Beijing. Excel spreadsheets (Microsoft) were used to generate random allocation sequences for groups. Participants were randomly divided into control, intervention 1 and intervention 2 groups, with 33 participants/group. The intervention lasted 13 weeks. Interventions are outlined (Table 1).

**Table 1:** Groups and interventions

	Week 1–4	Week 5–13
**Control group**	1) Daily dietary energy intake 800–1500 kcal. 2) Daily carbohydrate intake 50–150 g. 3) Daily staple food intake < 200 g. 4) Recommended ≥ 6000 steps/day.	
**Intervention group 1**	1) Daily dietary energy intake 800–1500 kcal. 2) Daily carbohydrate intake 50–150 g. 3) 38 g nutritional protein powder for dinner instead of staple foods. 4) Total staple food intake for breakfast and lunch < 150 g. 5) 6 g dietary fiber and 1.5 g fish oil added daily. 6) Recommended ≥ 6000 steps/day.	1) Daily dietary energy intake 800–1500 kcal. 2) Daily carbohydrate intake 50–150 g. 3) Have 38 g nutritional protein powder for breakfast and dinner to replace staple food. 4) Lunch staple food intake < 100 g. 5) 12 g dietary fiber and 3 g fish oil added daily. 6) Recommended ≥ 6000 steps/day.
**Intervention group 2**	1) Daily dietary energy intake 800–1500 kcal. 2) Daily carbohydrate intake 50–150 g. 3) 38 g nutritional protein powder for breakfast and dinner to replace staple foods. 4) Staple food intake for lunch < 100 g. 5) 12 g dietary fiber and 3 g fish oil added daily. 6) Recommended ≥ 6000 steps/day.	

Note: Protein nutritional powder is rich in soybean protein and provides 664 kJ energy/38 g: 20.1 g protein, 5.7 g fat, and 5.4 g carbohydrate. Products provided by Shanghai Nature’s Sunshine Health Products Co., Ltd.

### Ethics approval

The study was approved by the Ethics Committee of China Clinical Registration Trials (ethics review number: ChiECRCT20200292 and clinical registration number: ChiCTR2100050070). Participants signed consent forms before study commencement.

### Test indices

Before inclusion and after 4 and 13 weeks of the intervention, participants underwent physical examinations.

#### Before the intervention

(1) Physical examination: height, weight, waist circumference, hip circumference, and blood pressure. (2) Blood indicators: blood glucose, blood lipids, liver function, kidney function, and inflammation risk indicators. (3) DEXA analysis to assess body fat.

#### Four weeks into the intervention

1). Physical examination as described. 2) Blood indicators as described.

#### Thirteen weeks into the intervention

(1) Physical examination as described; (2) Blood indicators as described plus cell adipose indicators. (3) DEXA analysis as described; (4) Feces collection for intestinal flora analyses.

### Statistical analyses

We used SPSS 23.0 software to process and analyze data. If data conformed to a normal distribution, they were expressed as the mean ± standard deviation (X ± S). If data had a skewed distribution, they were expressed as the median ± interquartile interval (Me ± IQR). For data conforming to normality and variance homogeneity, single factor analysis of variance was used for comparisons between groups. For data not conforming to normality or variance homogeneity, rank sum tests were used. A *P* < 0.05 value was considered statistically significant.

## Results

### Flow chart

**Fig. 1:**
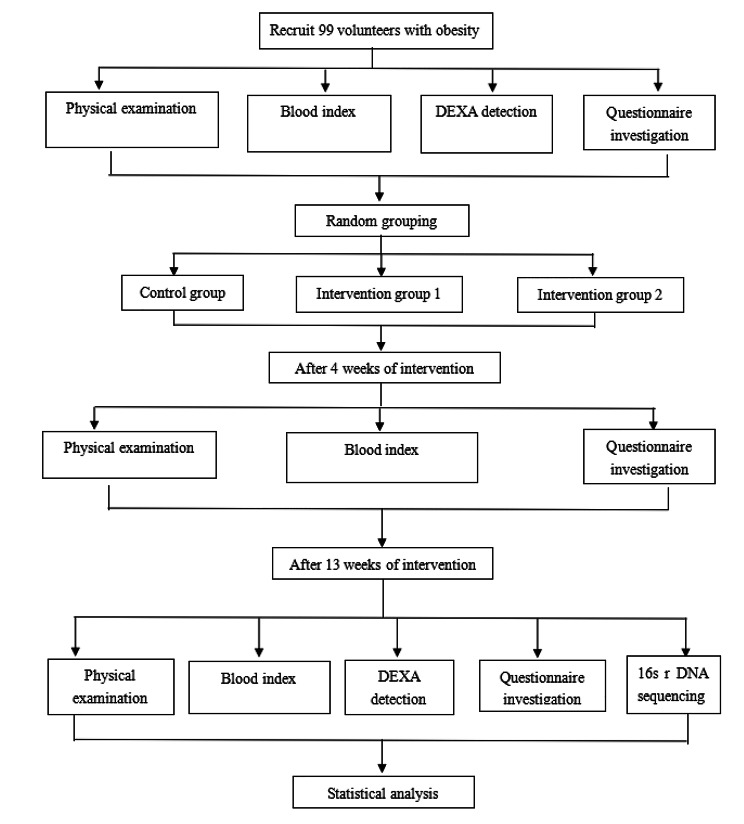
Study flow chart

A study flow chart is shown (Fig. 1).

### Participant compliance and loss to follow-up

In this study, 99 participants who met inclusion and exclusion criteria were randomized into three study groups, with 33/group. During follow-up, four participants in the control group failed to participate due to personal reasons; loss rate = 12.12%. Four participants in intervention group 1 were lost to follow-up due to personal reasons; follow-up rate = 12.12%. Two participants in intervention group 2 were lost to follow-up due to personal reasons, one to diarrhea and two to gout attack; loss rate = 15.15%. No statistical differences in follow-up loss rates were recorded between groups (Chi square test and *P* > 0.05).

### Participant characteristics

Data analyses showed that participant traits such as age, gender, nationality, labor intensity, education level, marital status, smoking, drinking, and height were balanced and comparable, with no statistical differences between groups (Table 2).

**Table 2:** Participant baseline data

	Control group	Intervention group 1	Intervention group 2	*P* (total)
Age ^a^	33.00 ± 14.00	34.00 ± 11.00	33.00 ± 14.00	0.657
Gender ^b^				0.214
male	26	25	20	
female	7	8	13	
Nationality ^b^				0.384
Han	33	32	32	
others	0	1	1	
Labor intensity ^b^				0.169
light manual labor	31	29	28	
medium manual labor	2	4	4	
heavy physical labor	0	0	1	
Education level ^b^				0.088
bachelor degree or above	23	28	28	
junior college	4	2	2	
high school or technical secondary school	3	1	3	
junior high school	2	2	0	
primary school and below	1	0	0	
Marital status ^b^				0.094
married	25	24	19	
unmarried	8	8	13	
divorce	0	1	1	
Smoking situation ^b^				0.223
no smoking	26	29	23	
used to smoke and have quit smoking	2	0	1	
smoke	5	4	9	
Drinking situation ^b^				0.119
no drinking	28	22	22	
used to drink and have quit smoking	1	0	2	
drink	4	11	9	
Height (cm) ^a^	171.60 ± 11.90	173.30 ± 16.60	170.20 ± 14.70	0.907

^a^Skewness distribution was expressed by the median ± interquartile range (Me ± IQR). The rank sum test was used to test for comparisons between groups

^b^Classified variables were expressed by frequency. Chi square tests were used to test for comparisons between groups

### Participant dietary status before and after intervention

Participant diets across all groups are shown (Table 3). At baseline, the daily intake of energy, carbohydrate, protein, and fat across groups was similar, with no statistical differences. During the initial 1–4 week intervention, when compared with controls, the daily carbohydrate intake in intervention groups was significantly lower when compared with controls. The daily protein intake of intervention group 1 was higher when compared with controls, but no statistical differences were recorded. The daily protein intake of intervention group 2 was significantly higher when compared with controls. During the 5–13 week intervention, when compared with controls, the daily carbohydrate intake of intervention groups was significantly lower when compared with controls. Daily protein intake was significantly higher when compared with controls.

**Table 3:** Participant dietary status before and after intervention

		Control group	Intervention group 1	Intervention group 2	*P* (total)	*P*(Intervention group 1/ Control group)	*P*(Intervention group 2/ Control group)	*P*(Intervention group 1/ Intervention group 2)
Energy (kcal/d)	Baseline ^a^	1616.05 ± 441.52	1751.99 ± 386.98	1738.05±350.35	0.547	0.320	0.372	0.918
	1–4 week ^b^	1270.57±166.57	1230.14±226.83	1306.36±259.18	0.462	0.461	0.461	0.262
	5–13 week ^c^	1348.00±253.60	1312.50±239.50	1356.00±275.00	0.330	0.883	0.156	0.256
Carbohydrate (g/d)	Baseline ^a^	213.92±66.35	227.74±58.87	234.43±63.32	0.628	0.525	0.347	0.758
	1–4 week ^b^	144.50±12.80	124.11±33.68	117.97±33.45	**< 0.001**	**0.005**	**< 0.001**	1.000
	5–13 week ^c^	148.10±20.30	124.10±36.40	122.20±50.20	**< 0.001**	**< 0.001**	**0.002**	1.000
Protein (g/d)	Baseline ^c^	79.10±35.50	74.30±31.00	63.20±38.20	0.831	0.796	0.547	0.730
	1–4 week ^c^	68.20±22.30	77.90±31.20	89.90±25.20	**< 0.001**	0.729	**< 0.001**	**0.015**
	5–13 week ^c^	67.70±26.90	91.20±18.80	92.20±18.30	**< 0.001**	**0.002**	**< 0.001**	1.000
Fat (g/d)	Baseline ^a^	50.97±29.58	60.19±27.42	56.61±24.42	0.612	0.328	0.549	0.703
	1–4 week ^c^	46.60±16.60	49.70±23.60	48.50±32.70	0.928	0.903	0.783	0.715
	5–13 week ^a^	50.27±13.56	48.70±13.83	53.10±17.77	0.493	0.674	0.452	0.242

^a^Normal variance was homogeneous and expressed by the mean ± standard deviation (X ± S). Single factor analysis of variance was used for comparisons between groups

^b^When normal variance was uneven, it was expressed by the mean ± standard deviation (X ± S). The rank sum test was used for inter-group comparisons

^c^Skewness distribution was expressed by the median ± interquartile interval (Me ± IQR). The rank sum test was used for comparisons between groups

### Participant changes in anthropometric indicators before and after intervention

Changes in anthropometric indicators across groups at baseline, weeks 4 and 13 of the intervention, and also 4- and 13-week variations are shown (Table 4). At baseline, weight, BMI, waist circumference, hip circumference, systolic blood pressure, and diastolic blood pressure across groups were similar, with no statistical differences.

After week 13, waist circumference, hip circumference and systolic blood pressure in controls had decreased, while weight, BMI, and diastolic blood pressure increased slightly. When compared with controls, body weight, BMI, waist circumference, hip circumference, systolic blood pressure, and diastolic blood pressure values in intervention group 1 were significantly reduced. When compared with controls, weight, BMI, waist circumference, and hip circumference values in intervention group 2 were significantly reduced. When compared with controls, systolic and diastolic blood pressure in intervention group 2 had decreased, but no statistical differences were recorded.

**Table 4:** Participant anthropometric indicator changes before and after intervention

		Control group	Intervention group 1	Intervention group 2	*P* (total)	*P*(Intervention group 1/ Control group)	*P*(Intervention group 2/ Control group)	*P*(Intervention group 1/ Intervention group 2)
Weight (kg)	Baseline ^a^	90.10 ± 10.62	88.12 ± 8.51	89.71 ± 12.31	0.724	0.449	0.880	0.544
	Week 4 ^a^	90.68 ± 9.94	86.62 ± 9.06	88.09 ± 12.12	0.283	0.118	0.317	0.568
	Week 13 ^a^	90.88 ± 9.76	85.02 ± 9.38	87.15 ± 11.92	0.074	**0.024**	0.149	0.407
	4-week variation ^a^	-0.16 ± 1.64	-1.50 ± 2.37	-2.19 ± 2.08	**< 0.001**	**0.009**	**< 0.001**	0.174
	13 week variation ^b^	0.20 ± 4.00	-4.00 ± 5.00	-1.90 ± 3.40	**< 0.001**	**< 0.001**	**0.003**	0.731
BMI(kg/m^2^)	Baseline ^b^	30.20 ± 3.80	30.53 ± 2.80	30.50 ± 2.70	0.544	0.873	0.480	0.251
	Week 4 ^b^	30.80 ± 4.50	29.40 ± 2.90	30.10 ± 2.70	0.203	0.098	0.296	0.323
	Week 13 ^a^	30.84 ± 2.47	29.29 ± 2.40	29.96 ± 2.05	**0.027**	**0.007**	0.123	0.240
	4-week variation ^a^	-0.04 ± 0.55	-0.52 ± 0.81	-0.76 ± 0.69	**< 0.001**	**0.006**	**< 0.001**	0.159
	13 week variation ^b^	0.10 ± 1.30	-1.40 ± 1.80	-0.70 ± 1.30	**< 0.001**	**< 0.001**	**0.002**	0.740
Waist circumference (cm)	Baseline ^c^	101.42 ± 7.51	102.41 ± 4.93	102.30 ± 7.78	0.806	0.485	0.677	0.969
	Week 4 ^a^	100.47 ± 7.10	99.72 ± 5.68	99.17 ± 8.70	0.766	0.674	0.468	0.760
	Week 13 ^a^	99.63 ± 7.38	94.96 ± 6.20	96.56 ± 9.51	0.052	**0.017**	0.114	0.408
	4-week variation ^a^	-0.97 ± 3.58	-2.69 ± 4.77	-3.38 ± 4.60	0.073	0.111	**0.027**	0.522
	13 week variation ^b^	-1.00 ± 5.40	-8.00 ± 7.40	-7.10 ± 8.00	**< 0.001**	**< 0.001**	**0.002**	1.000
Hip circumference (cm)	Baseline ^a^	109.27 ± 5.96	109.60 ± 4.49	110.44 ± 5.28	0.651	0.798	0.370	0.521
	Week 4 ^a^	109.23 ± 5.66	108.09 ± 5.32	107.59 ± 4.40	0.421	0.374	0.200	0.693
	Week 13 ^a^	109.12 ± 5.72	105.10 ± 4.69	106.84 ± 5.53	**0.011**	**0.003**	0.085	0.188
	4-week variation ^a^	-0.49 ± 3.39	-1.51 ± 4.33	-2.60 ± 4.63	0.126	0.324	**0.042**	0.288
	13 week variation ^a^	-0.36 ± 3.51	-5.13 ± 5.02	-3.21 ± 4.78	**< 0.001**	**< 0.001**	**0.011**	0.085
Systolic blood pressure (mm Hg)	Baseline ^b^	133.00 ± 15.00	130.00 ± 19.00	123.00 ± 21.00	0.257	0.663	0.133	0.202
	Week 4 ^b^	128.00 ± 16.00	127.00 ± 18.00	126.00 ± 13.00	0.937	0.672	0.873	0.954
	Week 13 ^b^	133.00 ± 11.00	125.00 ± 15.00	124.00 ± 14.00	**0.018**	**0.034**	**0.051**	1.000
	4-week variation ^b^	-4.00 ± 12.00	-6.00 ± 13.00	0.00 ± 16.00	0.221	0.480	0.296	0.088
	13 week variation ^b^	-1.00 ± 14.00	-5.00 ± 14.00	-4.00 ± 14.00	**0.048**	**0.044**	0.404	1.000
Diastolic pressure (mm Hg)	Baseline ^b^	84.00 ± 16.00	87.00 ± 25.00	81.00 ± 18.00	0.392	0.530	0.430	0.182
	Week 4 ^b^	85.00 ± 15.00	85.00 ± 16.00	83.00 ± 12.00	0.432	0.878	0.275	0.255
	Week 13 ^b^	86.00 ± 12.00	82.00 ± 7.00	84.00 ± 11.00	0.180	0.165	0.073	0.903
	4-week variation ^b^	-1.00 ± 7.00	-2.00 ± 10.00	-2.00 ± 11.00	0.619	0.500	0.738	0.352
	13 week variation ^b^	1.00 ± 9.00	-3.00 ± 12.00	-1.00 ± 4.00	**0.023**	**0.018**	0.419	0.618

^a^Normal variance was homogeneous and expressed by the mean ± standard deviation (X ± S). Single factor analysis of variance was used for comparisons between groups

^b^Skewness distribution was expressed by the median ± interquartile interval (Me ± IQR). Rank sum tests were used for comparisons between groups

^c^If the normal variance is uneven, it is expressed by the mean ± standard deviation (X ± S). Rank sum tests were used for inter-group comparisons

### Participant fat rate changes before and after intervention

Fat rate changes across groups at baseline, week 13, and 13-week variations are shown (Table 5). At baseline, whole body, head, left upper limb, right upper limb, trunk, left lower limb, and right lower limb fat rates across groups were similar, with no significant differences. After week 13, when compared with controls, body, head, left upper limb, trunk, and right lower limb fat rates in intervention group 1 decreased, but no statistical differences were recorded. When compared with controls, total body, head, left upper, trunk, left lower limb, and right lower limb fat rates in intervention group 2 was decreased, but no statistical differences were recorded. During the intervention period, the lean tissue content of the three groups of subjects changed slightly, and there was no significant decrease.

### Participant blood glucose and blood lipid index changes before and after intervention

Changes in blood glucose and blood lipid indices across groups at baseline and weeks 4 and 13, and also 4- and 13-week variations are shown (Table 6). At baseline, fasting blood glucose, glycosylated hemoglobin, glycosylated albumin, fasting insulin, triglyceride (TG), total cholesterol, high-density lipoprotein cholesterol (HDL-c), LDL-c, apolipoprotein A1, and apolipoprotein B levels across groups were similar, with no statistical differences.After week 13, fasting blood glucose, glycosylated albumin, fasting insulin, total cholesterol, HDL-c, and LDL-c levels in controls decreased, while HbA1c was unchanged. TG, apolipoprotein A1, and apolipoprotein B levels increased slightly. Furthermore, fasting blood glucose, glycosylated hemoglobin, glycosylated albumin, fasting insulin, TG, total cholesterol, HDL-c, LDL-c, and apolipoprotein B levels in intervention group 1 decreased, while apolipoprotein A1 increased slightly.

Fasting blood glucose, glycosylated albumin, fasting insulin, TG, total cholesterol, and HDL-c levels were decreased in intervention group 2, while HbA1c remained unchanged. LDL-c, apolipoprotein A1, and apolipoprotein B levels increased slightly. Among variables, when compared with controls, TGs in both groups were significantly reduced. When compared with controls, fasting blood glucose, glycosylated hemoglobin, glycosylated albumin, total cholesterol, and apolipoprotein B levels in intervention group 1 decreased, but no statistical differences were observed. When compared with controls, HDL-c in intervention group 1 decreased slightly, with no statistical differences.When compared with controls, glycosylated albumin, TG, and total cholesterol levels in intervention group 2 decreased, but no statistical difference was observed. When compared with controls, HDL-c in intervention group 2 decreased slightly, with no statistical differences.

### Participant liver and kidney function indices before and after intervention

Changes in liver and kidney function indices across groups at baseline and weeks 4 and 13, and also 4- and 13-week variations are shown (Table 7). At baseline, total bilirubin, alanine aminotransferase, aspartate aminotransferase, alkaline phosphatase, lactate dehydrogenase, urea, and creatinine levels across groups were similar, with no statistical differences. After week 13, when compared with controls, total bilirubin in the intervention group 1 had increased significantly but remained in the normal range with no clinical significance. Importantly, liver and kidney function indices showed no significant changes with no statistical differences during the intervention.

### Participant inflammatory risk, adipocytokines, and other indicators before and after intervention

Inflammation risk and adipocyte factor indicators across groups are shown (Table 8). After week 13, high sensitive C-reactive protein (CRP), myeloperoxidase (MPO), oxidized LDL (Ox-LDL), leptin (LEP), transforming growth factor-β_1_ (TGF-β_1_), interleukin-6 (IL-6), glycosylphosphatidylinositol specific phospholipase D1 (GPLD1), preneurotensin (pro NT), recombinant glypican 4 (GPC-4), and lipopolysaccharide (LPS) in both intervention groups were lower when compared with controls, but no statistical differences were recorded. Adiponectin (ADPN) levels in both groups were higher when compared with controls, with no statistical differences. Tumor necrosis factor-α (TNF- α) in intervention group 1 was lower when compared with controls, with no statistical differences. 

### Fecal intestinal flora analyses

#### Differences between groups

##### Phyla

**Fig. 2:**
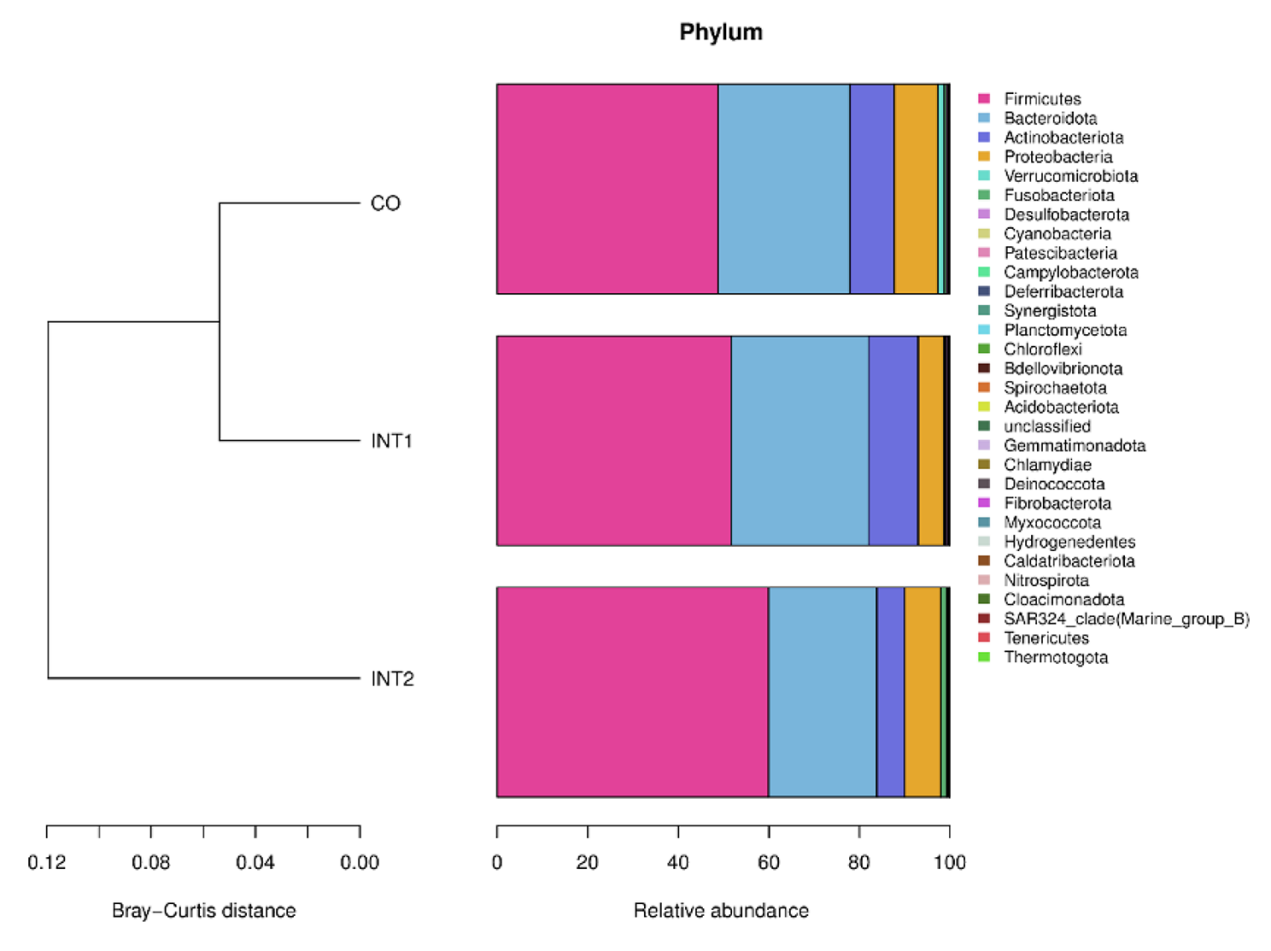
Phyla structures across groups

**Fig. 3:**
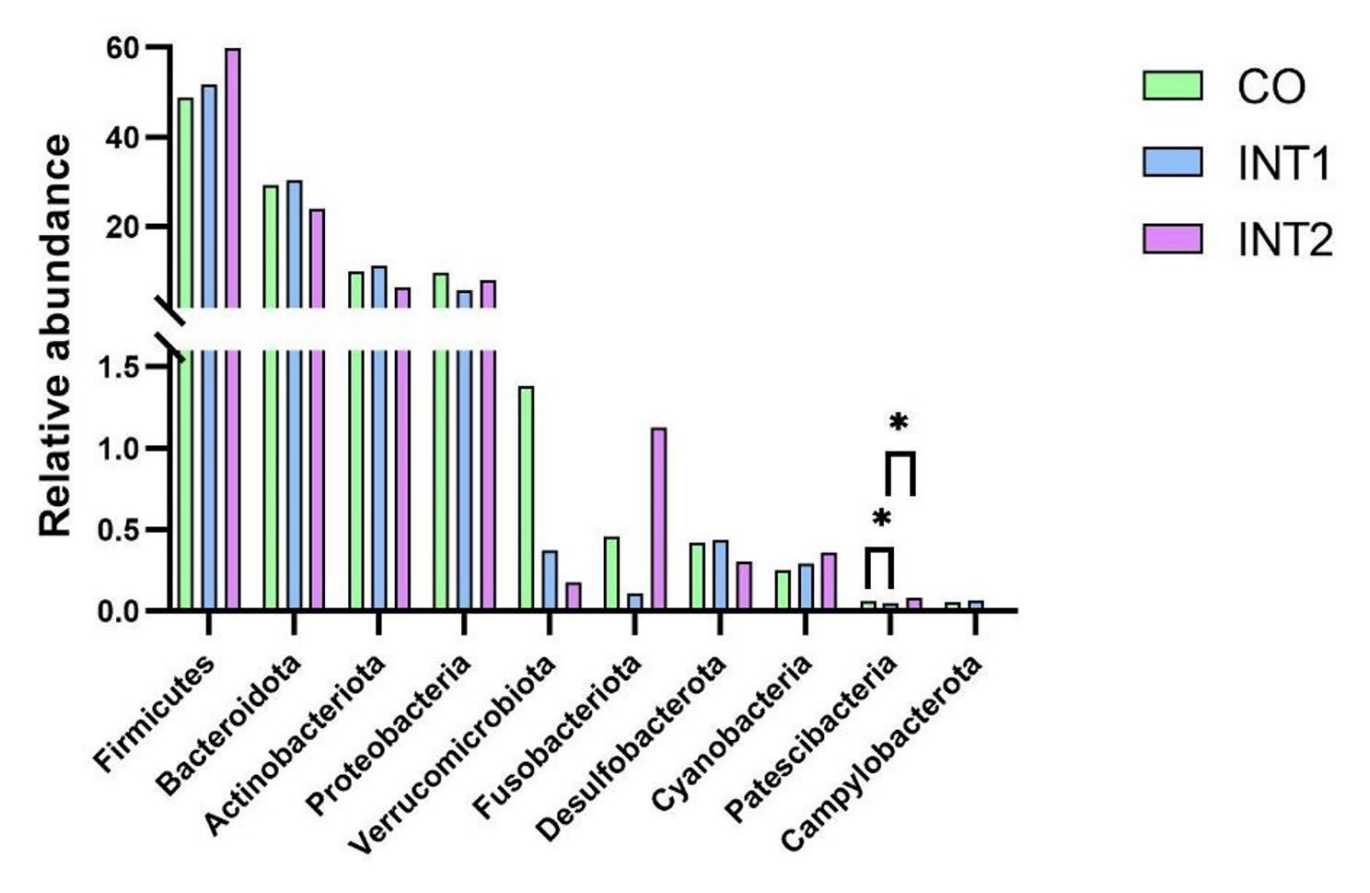
Comparing phyla across groups

As shown (Fig. 2), the top 10 phyla in intestinal flora across groups were *Firmicutes, Bacteroidota, Actinobacteria, Proteobacteria, Verrucomicrobiota, Fusobacteriota, Desulfobacterota, Cyanobacteria, Patescibacteria*, and *Campylobacterota*, of which, *Firmicutes, Bacteroidota*, and *Actinobacia* were dominant. As shown (Fig. 3), among the top 10 bacteria, except that the Patescibacteria in the control group and the intervention group 2 were significantly higher than that in the intervention group 1 (P < 0.05), the other nine kinds of bacteria had no statistical difference among the three groups. Thus, on the whole, no significant phyla differences were observed among groups.

##### Genera

**Fig. 4:**
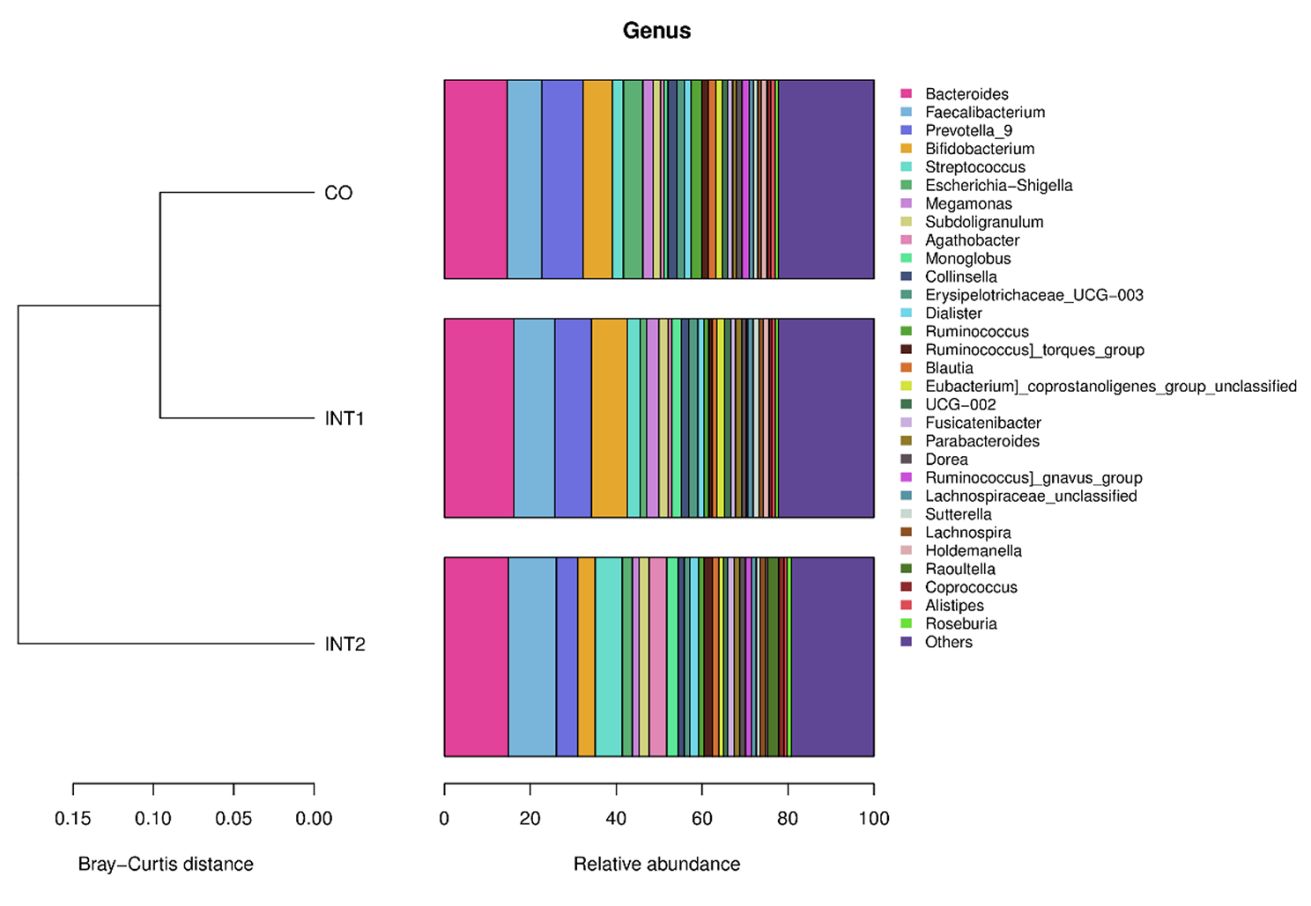
Genera structural composition across groups

**Fig. 5:**
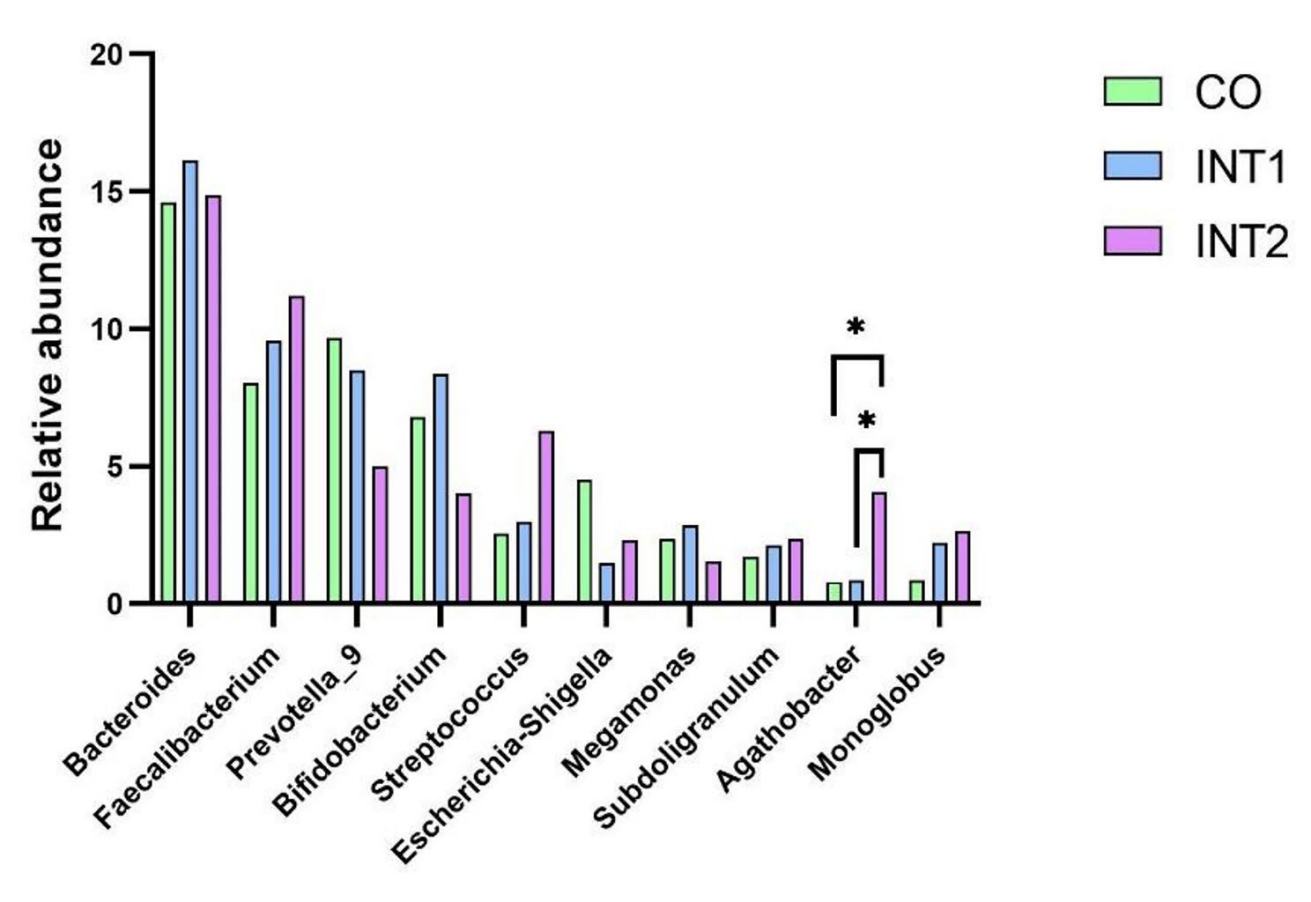
Comparing genera across groups

As indicated (Fig. 4), the top 10 genera across groups were *Bacteroides, Faecalibacterium, Prevotella_9, Bifidobacterium, Streptococcus, Escherichia-Shigella, Megamonas, Subdoligranulum, Agathobacter*, and *Monoglobus*, of which, *Bacteroides, Faecalibacterium*, and *Prevotella_9* were dominant. As shown (Fig. 5), among the top 10 bacterial species, except that the *Agathobacter* in the intervention group 2 was significantly higher than that in the control group and the intervention group 1 (P < 0.05), there was no statistical difference among the other nine species of bacteria in the three groups. Therefore, on the whole, no significant differences in genera were identified among groups.

## Discussion

After a 13 week dietary intervention, when compared with controls, body weight, BMI, waist circumference, hip circumference, systolic blood pressure, and diastolic blood pressure values in intervention group 1 were statistically significantly reduced. Body weight, BMI, waist circumference, and hip circumference in intervention group 2 were statistically significantly reduced. TGs in both groups were statistically significantly reduced.

Typically, LCDs have a carbohydrate energy supply ratio ≤ 40%, fat energy supply ratio ≥ 30%, relatively increased protein levels, and limited or unlimited total energy intake [10, 11]. Individuals on LCDs tend to have faster weight loss [12]. Sun et al. [13] examined the combination of energy unlimited LCD and exercise to intervene for 4 weeks in overweight Chinese women and found that participant weight, waist circumference, and hip circumference were significantly reduced.

Food substitutes can generate sustainable weight-loss effects by reducing food types and controlling food portions, thereby improving obesity-related disease risk factors and minimizing lean weight loss so as to maintain strength, physical function, and weight over extended periods [14]. System evaluations also showed that weight-loss effects, by substituting foods, improved within 1 year, and may be used as effective obesity management strategies for community and health care institutions [15]. Also, dietary substitution was effective for managing obesity and type 2 diabetes [16–18]; the approach improved fat quality, blood pressure, glycosylated hemoglobin, insulin, and other indicators [19, 20].

A recent systematic review and meta-analysis reported that in obese patients (BMI = 36–43 kg/m²), weight loss was 8.9–15.0 kg after very low (< 800 kcal/day) or low calorie (> 800 kcal/day) liquid-meal substitutes were used [21]. We hypothesize that weight-loss differences in this meta-analysis were possibly due to differences in daily calorie intake. Studies have shown that moderate and sustained weight loss reduces the risk of long-term adverse outcomes when compared with rapid weight loss [22]. Therefore, in our study, we selected a more modest daily energy intake target (800–1500 kcal/day), which increased participant compliance and reduced follow-up losses.

Research has also shown that when compared with traditional energy-limiting diets, soybean protein-based energy limiting diets significantly reduce serum TC and LDL-C levels and body fat content in overweight adults [8]. A soybean protein based high-protein diet is more acceptable and improves weight loss, body composition, and cardiac health indicators [23]. In our study, we explored differences between an LCD (reduced staple food intake) with an LCD (some staple foods replaced with nutritional protein powder (soybean protein)) while supplementing with dietary fiber and fish oil. After week 13, when compared with controls, both intervention groups showed significant weight loss and reduced blood pressure and blood lipids, consistent with Clinton et al. [24]. Thus, our LCD (staple food replacement with nutritional protein powder and dietary fiber and fish oil) had greater effects on weight loss, blood pressure, and blood lipid reduction, amongst others.

Obesity is a complex multifactorial disease and is defined as “abnormal or excessive fat accumulation in adipose tissue” [25, 26]. Adipose tissue is important for energy storage and endocrine and immune regulation. Many cytokines, hormones, extracellular matrix proteins, and other bioactive factors are synthesized and released by adipose tissue and are known as adipokines [27]. To a large extent, adipose tissue dysfunction in obese patients is manifested by imbalanced proinflammatory and anti-inflammatory adipose factor expression [28], which initiates chronic inflammation in adipose tissue and causes insulin resistance and multiple metabolic disorders [27]. Research now shows that TNF-α and IL-6 are fat factors which induce insulin resistance [29, 30]. ADPN is also a fat factor which improves insulin resistance [31]. Research also indicates that adipose tissue is a target tissue of ADPN, which increases insulin sensitivity and resists macrophage infiltration and inflammatory factor expression caused by obesity [32]. ADPN also inhibits TNF-α in many cell types and exerts anti-inflammatory effects [33]. GPLD1 is a phospholipase which cleaves GPI [34] and may cleave GPC4 [35]. Serum GPC4 levels are speculated to be positively correlated with BMI and body fat levels [36, 37]. Our study data are generally consistent with the literature.

Current evidence also suggests close relationships between intestinal flora and obesity, with intestinal microorganisms having important roles in food digestion and metabolic regulation [38, 39]. Intestinal flora metabolic activity impacts nutrient absorption, which in turn affects energy balance during energy storage and consumption by promoting the energy metabolism of dietary components [40, 41]. We showed that intestinal flora levels across all study groups exhibited α- and β-diversity, but no distinct diversity differences were recorded. In the top 10 bacterial species, except for Patescibacteria in controls and intervention group 2, which were significantly higher than intervention group 1 (P < 0.05), the remaining nine bacterial species showed no statistical differences. Among the first 10 species of bacteria, except that the *Agathobacter* in the intervention group 2 was significantly higher than that in the control group and the intervention group 1 (P < 0.05), no statistical differences were identified in the remaining nine bacterial species across groups. Dietary fiber are edible carbohydrate polymers comprising three or more monomeric units are resistant to endogenous digestive enzymes and are neither hydrolyzed nor absorbed in the small intestine [42]. *Firmicutes* and *Actinomycetes* are the main dietary fiber responders [43]. In our intervention groups, we replaced some staple foods with nutritional protein powder (soybean protein) and dietary fiber and fish oil at the same time. However, our small sample size, large individual differences, and short intervention times may have contributed to many non-significant differences in our data.

After 4 weeks, weight loss and metabolic improvement effects in intervention group 2 were much better when compared with intervention group 1. But after week 13, intervention group 1 effects were better when compared with intervention group 2. Nutritional protein powder, dietary fiber, and fish oil doses in intervention group 1 in the first 4 weeks were low, but doubled in the next 9 weeks. The total intake in intervention group 2 in 13 weeks was the same as intervention group 1 in the later 9 weeks. Thus, a dose-doubling intervention for intervention group 1 improved participant compliance and weight loss effects.

Our study had some limitations. First, the sample size was small. Second, we did not conduct regular follow-up checks after the study to assess diet sustainability. Finally, as the weight-loss intervention period spanned autumn/winter, the weather was getting colder, thus if a spring/ summer study was conducted, weight-loss effects may have improved.

## Conclusions

We showed that an LCD, where nutritional protein powder was used to replace some staple foods, and dietary fiber and fish oils were simultaneously supplemented, significantly reduced weight and improved carbohydrate and lipid metabolism in obese individuals was observed when compared with an LCD that reduced staple food intake. Our study provides a scientific and healthy nutritional intervention for obese individuals who wish to lose weight.

## Electronic supplementary material

Below is the link to the electronic supplementary material.

Supplementary Material 1 Tables

## Data Availability

All data generated or analyzed during this study are included in this published article.
