# Correction: The effects of a low carbohydrate diet combined with partial meal replacement on obese individuals

**DOI:** 10.1186/s12986-023-00747-y

**Published:** 2023-05-10

**Authors:** Yulian Zhong, Ximin Chen, Chao Huang, Yuexiao Chen, Fengyi Zhao, Runhua Hao, Niannian Wang, Wang Liao, Hui Xia, Ligang Yang, Shaokang Wang, Guiju Sun

**Affiliations:** 1 Key Laboratory of Environmental Medicine and Engineering of Ministry of Education, Department of Nutrition and Food Hygiene, School of Public Health, Southeast University, Nanjing, 210009 Jiangsu China; 2 Beijing Institute of Nutritional Resources, Beijing, 100069 China; 3 Institute of Biotechnology and Health, Beijing Academy of Science and Technology, Beijing, 100089 China

## Correction: Nutrition & Metabolism (2023) 20:18 10.1186/s12986-023-00740-5

Following publication of the original article [1], the authors identified an error in Table 1. The correct table is given below.

**Table 1:** Groups and interventions

	Week 1–4	Week 5–13
Control group	1) Daily dietary energy intake 800–1500 kcal. 2) Daily carbohydrate intake 50–150 g. 3) Daily staple food intake < 200 g. 4) Recommended ≥ 6000 steps/day.	
Intervention group 1	1) Daily dietary energy intake 800–1500 kcal. 2) Daily carbohydrate intake 50–150 g. 3) 38 g nutritional protein powder for dinner instead of staple foods. 4) Total staple food intake for breakfast and lunch < 150 g. 5) 6 g dietary fiber and 1.5 g fish oil added daily. 6) Recommended ≥ 6000 steps/day.	1) Daily dietary energy intake 800–1500 kcal. 2) Daily carbohydrate intake 50–150 g. 3) Have 38 g nutritional protein powder for breakfast and dinner to replace staple food. 4) Lunch staple food intake < 100 g. 5) 12 g dietary fiber and 3 g fish oil added daily. 6) Recommended ≥ 6000 steps/day.
Intervention group 2	1) Daily dietary energy intake 800–1500 kcal. 2) Daily carbohydrate intake 50–150 g. 3) 38 g nutritional protein powder for breakfast and dinner to replace staple foods. 4) Staple food intake for lunch < 100 g. 5) 12 g dietary fiber and 3 g fish oil added daily. 6) Recommended ≥ 6000 steps/day.	

Note: Protein nutritional powder is rich in soybean protein and provides 664 kJ energy/38 g: 20.1 g protein, 5.7 g fat, and 5.4 g carbohydrate. Products provided by Shanghai Nature's Sunshine Health Products Co., Ltd

The original article [1] has been corrected.
