# Supplementary material for: The effects of a low carbohydrate diet combined with partial meal replacement on obese individuals
Source: Nutr Metab (Lond). 2023 Mar 30;20:18. doi: 10.1186/s12986-023-00740-5 (PMC10064565; doi:10.1186/s12986-023-00740-5)
Supplement: Supplementary file 1 — Supplementary Material 1 Tables [file 12986_2023_740_MOESM1_ESM.docx]

**Table 5. Participant fat rate changes before and after intervention**

|  |  | Control group | Intervention group 1 | Intervention group 2 | *P* (total) | *P*(Intervention group 1/ Control group) | *P*(Intervention group 2/ Control group) | *P*(Intervention group 1/ Intervention group 2) |
| --- | --- | --- | --- | --- | --- | --- | --- | --- |
| Body fat rate (%) | Baseline ^a^ | 36.16±5.02 | 36.61±5.15 | 37.51±5.47 | 0.565 | 0.726 | 0.295 | 0.485 |
|  | Week 13 ^a^ | 34.84±4.89 | 34.73±5.19 | 36.07±4.16 | 0.452 | 0.928 | 0.297 | 0.258 |
|  | 13 week variation ^b^ | -0.80±2.90 | -2.20±2.90 | -2.10±2.70 | 0.140 | 0.797 | 0.178 | 0.191 |
| Head fat rate (%) | Baseline ^b^ | 22.80±1.60 | 22.90±1.80 | 23.00±1.40 | 0.691 | 0.883 | 0.437 | 0.488 |
|  | Week 13 ^b^ | 22.70±1.20 | 22.80±1.60 | 23.00±1.30 | 0.827 | 0.768 | 0.630 | 0.603 |
|  | 13 week variation ^b^ | 0.00±0.20 | -0.10±0.30 | -0.20±0.20 | 0.055 | 0.553 | **0.019** | 0.093 |
| Fat rate of left upper limb (%) | Baseline ^a^ | 40.69±9.00 | 40.85±8.18 | 44.39±8.53 | 0.145 | 0.938 | 0.082 | 0.096 |
|  | Week 13 ^a^ | 38.54±8.84 | 39.16±8.67 | 42.49±7.44 | 0.123 | 0.762 | 0.057 | 0.108 |
|  | 13 week variation ^b^ | -1.90±4.00 | -2.60±4.30 | -2.40±1.70 | 0.724 | 0.807 | 0.336 | 0.812 |
| Fat rate of right upper limb (%) | Baseline ^a^ | 40.15±8.52 | 39.69±8.32 | 43.38±8.73 | 0.165 | 0.827 | 0.127 | 0.082 |
|  | Week 13 ^a^ | 38.84±8.78 | 39.44±9.09 | 42.18±7.21 | 0.233 | 0.775 | 0.110 | 0.188 |
|  | 13 week variation ^b^ | -1.60±5.50 | -0.70±4.60 | -1.50±2.80 | 0.432 | 0.768 | 0.369 | 0.204 |
| Trunk fat rate (%) | Baseline ^b^ | 37.60±6.30 | 37.30±7.20 | 40.10±6.30 | 0.307 | 0.469 | 0.115 | 0.473 |
|  | Week 13 ^b^ | 37.50±6.10 | 36.00±5.50 | 37.60±7.50 | 0.516 | 0.857 | 0.329 | 0.317 |
|  | 13 week variation ^b^ | -1.00±3.10 | -2.90±3.50 | -2.20±3.10 | 0.058 | **0.022** | 0.480 | 0.099 |
| Fat rate of left lower limb (%) | Baseline ^a^ | 35.39±6.48 | 35.42±6.23 | 36.29±6.69 | 0.812 | 0.985 | 0.571 | 0.584 |
|  | Week 13 ^b^ | 33.30±8.50 | 34.00±7.90 | 34.00±5.30 | 0.512 | 0.586 | 0.233 | 0.594 |
|  | 13 week variation ^b^ | -0.90±2.80 | -0.30±3.30 | -1.50±3.30 | 0.648 | 0.529 | 0.368 | 0.758 |
| Fat rate of right lower limb (%) | Baseline ^a^ | 35.15±6.85 | 35.33±6.38 | 36.10±7.40 | 0.836 | 0.913 | 0.574 | 0.650 |
|  | Week 13 ^a^ | 33.44±6.45 | 33.73±5.98 | 34.80±5.66 | 0.630 | 0.845 | 0.362 | 0.473 |
|  | 13 week variation ^b^ | -1.40±3.30 | -1.80±2.90 | -1.70±3.20 | 0.360 | 0.158 | 0.964 | 0.296 |
| Fat tissue content (kg) | Baseline ^a^ | 32.4±5.5 | 32.1±4.1 | 33.0±6.2 | 0.789 | 0.827 | 0.649 | 0.501 |
|  | Week 13 ^a^ | 31.3±5.5 | 29.5±4.3 | 31.2±4.2 | 0.235 | 0.127 | 0.907 | 0.158 |
|  | 13 week variation ^a^ | -1.1±2.5 | -2.6±3.9 | -1.8±5.3 | 0.328 | 0.137 | 0.462 | 0.447 |
| Lean tissue content (kg) | Baseline ^a^ | 56.1±9.0 | 54.7±8.8 | 53.8±9.8 | 0.587 | 0.533 | 0.307 | 0.689 |
|  | Week 13 ^a^ | 57.4±8.3 | 54.7±9.0 | 54.7±9.9 | 0.380 | 0.230 | 0.228 | 0.996 |
|  | 13 week variation ^a^ | 1.2±4.1 | -0.1±2.1 | 0.8±4.8 | 0.389 | 0.182 | 0.688 | 0.349 |

^a^Normal variance was homogeneous and expressed by the mean ± standard deviation (X ± S). Single factor analysis of variance was used for comparisons between groups.

^b^Skewness distribution was expressed by the median ± interquartile interval (Me ± IQR). The rank sum test was used for comparisons between groups.

**Table 6. Participant blood glucose and blood lipid index changes before and after intervention**

|  |  | Control group | Intervention group 1 | Intervention group 2 | *P* (total) | *P*(Intervention group 1/ Control group) | *P*(Intervention group 2/ Control group) | *P*(Intervention group 1/ Intervention group 2) |
| --- | --- | --- | --- | --- | --- | --- | --- | --- |
| Fasting blood glucose (mmol/L) | Baseline ^a^ | 4.97±1.02 | 4.66±1.37 | 4.62±1.17 | 0.585 | 0.343 | 0.585 | 0.939 |
|  | Week 4 ^a^ | 4.64±0.56 | 4.78±0.67 | 4.91±0.92 | 0.072 | 0.264 | 0.072 | 0.195 |
|  | Week 13 ^a^ | 4.84±1.13 | 4.55±1.07 | 4.38±1.04 | 0.125 | **0.046** | 0.125 | 0.352 |
|  | 4 week variation ^a^ | -0.07±0.62 | -0.04±0.87 | 0.16±1.27 | **0.008** | 0.172 | **0.006** | 0.698 |
|  | 13 week variation ^a^ | -0.07±0.58 | -0.29±0.55 | -0.07±0.50 | 0.407 | 0.323 | 0.407 | 0.204 |
| Glycosylated hemoglobin (%) | Baseline ^a^ | 5.30±0.40 | 5.40±0.30 | 5.40±0.30 | 0.557 | 0.851 | 0.557 | 0.354 |
|  | Week 4 ^a^ | 5.40±0.50 | 5.30±0.30 | 5.50±0.30 | 0.449 | 0.974 | 0.449 | 0.272 |
|  | Week 13 ^a^ | 5.40±0.50 | 5.30±0.20 | 5.50±0.30 | **0.042** | 0.433 | 0.889 | **0.037** |
|  | 4 week variation ^a^ | 0.00±0.23 | -0.02±0.24 | 0.01±0.23 | 0.884 | 0.969 | 0.884 | 0.777 |
|  | 13 week variation ^a^ | 0.00±0.20 | -0.03±0.17 | 0.00±0.27 | 0.227 | 0.103 | 0.227 | 0.205 |
| Glycated albumin (%) | Baseline ^b^ | 12.88±1.41 | 13.06±1.05 | 12.70±1.08 | 0.471 | 0.537 | 0.541 | 0.221 |
|  | Week 4 ^b^ | 12.18±1.17 | 12.51±1.24 | 11.88±1.02 | 0.091 | 0.254 | 0.288 | **0.029** |
|  | Week 13 ^b^ | 12.67±1.25 | 12.58±0.87 | 12.39±0.95 | 0.542 | 0.722 | 0.279 | 0.465 |
|  | 4 week variation ^a^ | -0.70±1.77 | -0.31±2.30 | -0.81±2.20 | 0.533 | 0.868 | 0.359 | 0.314 |
|  | 13 week variation ^b^ | -0.25±0.62 | -0.51±0.49 | -0.30±0.49 | 0.107 | **0.047** | 0.697 | 0.108 |
| Fasting insulin (pmol/L) | Baseline ^a^ | 155.00±90.00 | 145.00±59.00 | 127.00±77.00 | 0.150 | 0.112 | 0.150 | 0.798 |
|  | Week 4 ^a^ | 128.00±47.00 | 110.00±55.00 | 114.00±33.00 | 0.051 | **0.030** | 0.051 | 0.339 |
|  | Week 13 ^a^ | 101.00±47.00 | 86.00±37.00 | 103.00±50.00 | 0.274 | 0.209 | 0.274 | 0.137 |
|  | 4 week variation ^a^ | -31.00±45.70 | -23.20±68.20 | -11.90±36.00 | 0.130 | 0.453 | 0.130 | 0.363 |
|  | 13 week variation ^a^ | -57.70±60.20 | -42.20±49.10 | -33.60±64.90 | 0.167 | 0.696 | 0.167 | 0.105 |
| Triglyceride (mmol/L) | Baseline ^a^ | 1.74±1.67 | 1.51±1.19 | 1.62±1.32 | 0.828 | 0.744 | 0.828 | 0.827 |
|  | Week 4 ^a^ | 1.74±0.79 | 1.26±0.90 | 1.56±0.93 | **0.010** | **0.007** | 0.295 | 0.507 |
|  | Week 13 ^a^ | 1.98±1.71 | 1.07±0.49 | 1.42±0.73 | **<0.001** | **<0.001** | **0.027** | 0.096 |
|  | 4 week variation ^a^ | -0.18±0.85 | -0.27±0.72 | -0.07±0.85 | 0.199 | 0.116 | 0.199 | 0.125 |
|  | 13 week variation ^a^ | 0.24±0.76 | -0.53±0.55 | -0.27±0.56 | **<0.001** | **<0.001** | **0.040** | 0.185 |
| Total cholesterol (mmol/L) | Baseline ^a^ | 4.74±1.35 | 4.99±1.35 | 4.94±1.16 | 0.867 | 0.705 | 0.867 | 0.608 |
|  | Week 4 ^b^ | 4.98±0.96 | 4.83±0.78 | 4.78±0.90 | 0.633 | 0.500 | 0.357 | 0.804 |
|  | Week 13 ^a^ | 4.98±0.91 | 4.78±1.17 | 5.00±1.32 | 0.567 | 0.290 | 0.567 | 0.803 |
|  | 4 week variation ^b^ | -0.16±0.48 | -0.30±0.59 | -0.30±0.64 | 0.525 | 0.311 | 0.344 | 0.947 |
|  | 13 week variation ^a^ | -0.08±0.76 | -0.18±0.42 | -0.10±0.46 | 0.703 | 0.630 | 0.703 | 0.379 |
| High density lipoprotein cholesterol (mmol/L) | Baseline ^b^ | 1.23±0.22 | 1.26±0.20 | 1.24±0.21 | 0.793 | 0.517 | 0.879 | 0.619 |
|  | Week 4 ^a^ | 1.02±0.13 | 1.16±0.24 | 1.08±0.27 | **0.019** | **0.018** | 1.000 | 0.175 |
|  | Week 13 ^a^ | 0.98±0.21 | 1.21±0.33 | 1.09±0.20 | **0.017** | **0.013** | 0.438 | 0.488 |
|  | 4 week variation ^a^ | -0.17±0.18 | -0.08±0.14 | -0.14±0.19 | 0.073 | **0.030** | 0.073 | 0.084 |
|  | 13 week variation ^a^ | -0.19±0.22 | -0.07±0.12 | -0.13±0.16 | **0.005** | **0.004** | 0.225 | 0.434 |
| Low density lipoprotein cholesterol (mmol/L) | Baseline ^a^ | 2.80±0.90 | 3.09±0.98 | 2.95±0.86 | 0.761 | 0.517 | 0.761 | 0.560 |
|  | Week 4 ^a^ | 3.07±0.97 | 3.07±0.64 | 2.86±1.09 | 0.639 | 0.893 | 0.639 | 0.356 |
|  | Week 13 ^b^ | 2.88±0.74 | 3.02±0.69 | 3.06±0.79 | 0.565 | 0.421 | 0.313 | 0.838 |
|  | 4 week variation ^a^ | 0.06±0.47 | -0.09±0.41 | -0.15±0.53 | 0.202 | 0.218 | 0.202 | 0.404 |
|  | 13 week variation ^a^ | -0.19±0.54 | -0.03±0.44 | 0.02±0.58 | 0.244 | 0.200 | 0.244 | 0.753 |
| Apolipoprotein A1 (g/L) | Baseline ^b^ | 1.25±0.16 | 1.32±0.21 | 1.26±0.17 | 0.274 | 0.136 | 0.819 | 0.206 |
|  | Week 4 ^a^ | 1.33±0.44 | 1.32±0.37 | 1.28±0.33 | 0.392 | 0.484 | 0.551 | 0.162 |
|  | Week 13 ^b^ | 1.36±0.23 | 1.34±0.22 | 1.31±0.26 | 0.731 | 0.755 | 0.433 | 0.636 |
|  | 4 week variation ^b^ | 0.03±0.19 | 0.01±0.20 | 0.00±0.15 | 0.803 | 0.731 | 0.509 | 0.752 |
|  | 13 week variation ^b^ | 0.09±0.16 | 0.02±0.16 | 0.05±0.19 | 0.270 | 0.107 | 0.407 | 0.428 |
| Apolipoprotein B (g/L) | Baseline ^b^ | 0.93±0.25 | 0.97±0.20 | 0.92±0.22 | 0.671 | 0.531 | 0.813 | 0.389 |
|  | Week 4 ^b^ | 1.01±0.24 | 0.93±0.17 | 0.95±0.25 | 0.311 | 0.139 | 0.287 | 0.675 |
|  | Week 13 ^c^ | 0.96±0.25 | 0.91±0.19 | 0.94±0.27 | 0.557 | 0.287 | 0.457 | 0.802 |
|  | 4 week variation ^a^ | 0.05±0.15 | -0.02±0.16 | -0.01±0.20 | **0.015** | **0.011** | 0.318 | 0.606 |
|  | 13 week variation ^b^ | 0.02±0.21 | -0.04±0.20 | 0.01±0.16 | 0.420 | 0.220 | 0.840 | 0.305 |

^a^Skewness distribution was expressed by the median ± interquartile interval (Me ± IQR). The rank sum test was used for comparisons between groups.

^b^Normal variance was homogeneous and expressed by the mean ± standard deviation (X ± S). Single factor analysis of variance was used for comparisons between groups.

^c^Normal variance was uneven and expressed by the mean ± standard deviation (X ± S). The rank sum test was used for inter-group comparisons.

**Table 7. Participant liver and kidney function indices before and after intervention**

|  |  | Control group | Intervention group 1 | Intervention group 2 | *P* (total) | *P*(Intervention group 1/ Control group) | *P*(Intervention group 2/ Control group) | *P*(Intervention group 1/ Intervention group 2) |
| --- | --- | --- | --- | --- | --- | --- | --- | --- |
| Total bilirubin (umol/L) | Baseline ^a^ | 12.70±8.30 | 12.10±8.60 | 11.00±7.30 | 0.654 | 0.744 | 0.369 | 0.551 |
|  | Week 4 ^a^ | 12.40±5.10 | 12.70±10.50 | 12.30±7.90 | 0.697 | 0.408 | 0.898 | 0.538 |
|  | Week 13 ^a^ | 12.30±6.60 | 14.50±9.10 | 11.40±6.20 | **0.043** | 0.150 | 1.000 | 0.058 |
|  | 4 week variation ^a^ | 0.80±8.70 | 1.90±7.90 | 1.10±10.20 | 0.434 | 0.287 | 0.246 | 0.847 |
|  | 13 week variation ^a^ | 0.30±8.80 | 2.60±8.70 | 0.90±9.00 | 0.055 | **0.025** | 0.137 | 0.216 |
| Alanine aminotransferase (U/L) | Baseline ^a^ | 24.00±28.00 | 23.00±27.00 | 23.00±33.00 | 0.946 | 0.758 | 0.913 | 0.807 |
|  | Week 4 ^a^ | 36.00±21.00 | 31.00±29.00 | 35.00±22.00 | 0.581 | 0.302 | 0.581 | 0.617 |
|  | Week 13 ^a^ | 28.00±18.00 | 24.00±16.00 | 24.00±18.00 | 0.777 | 0.529 | 0.551 | 0.933 |
|  | 4 week variation ^a^ | 2.00±23.00 | 3.00±16.00 | 5.00±23.00 | 0.824 | 0.985 | 0.635 | 0.551 |
|  | 13 week variation ^a^ | -4.00±24.00 | -1.00±21.00 | -2.00±16.00 | 0.903 | 1.000 | 0.827 | 0.590 |
| Aspartate aminotransferase (U/L) | Baseline ^a^ | 23.00±11.00 | 21.00±10.00 | 20.00±13.00 | 0.833 | 0.807 | 0.630 | 0.594 |
|  | Week 4 ^a^ | 21.00±8.00 | 22.00±8.00 | 21.00±8.00 | 0.968 | 0.928 | 0.782 | 0.903 |
|  | Week 13 ^a^ | 19.00±7.00 | 20.00±8.00 | 18.00±6.00 | 0.453 | 0.777 | 0.364 | 0.226 |
|  | 4 week variation ^a^ | -2.00±9.00 | -2.00±8.00 | 0.00±8.00 | 0.940 | 0.700 | 0.837 | 0.969 |
|  | 13 week variation ^b^ | -3.06±7.34 | -3.06±9.69 | -4.39±8.36 | 0.764 | 1.000 | 0.526 | 0.526 |
| Alkaline phosphatase (U/L) | Baseline ^c^ | 79.64±23.15 | 77.27±14.16 | 75.15±15.84 | 0.869 | 0.878 | 0.594 | 0.739 |
|  | Week 4 ^a^ | 77.00±26.00 | 75.00±25.00 | 75.00±17.00 | 0.646 | 0.390 | 0.468 | 0.837 |
|  | Week 13 ^a^ | 74.00±20.00 | 67.00±16.00 | 71.00±7.00 | 0.371 | 0.195 | 0.332 | 0.546 |
|  | 4 week variation ^a^ | 2.00±9.00 | 1.00±9.00 | 2.00±6.00 | 0.447 | 0.792 | 0.322 | 0.245 |
|  | 13 week variation ^a^ | -3.00±10.00 | -6.00±6.00 | -5.00±4.00 | 0.511 | 0.306 | 0531 | 0.432 |
| Lactate dehydrogenase (U/L) | Baseline ^a^ | 160.00±81.00 | 170.00±70.00 | 160.00±59.00 | 0.889 | 0.753 | 0.608 | 0.980 |
|  | Week 4 ^a^ | 212.00±52.00 | 198.00±35.00 | 181.00±38.00 | **0.002** | 0.749 | **0.002** | 0.061 |
|  | Week 13 ^a^ | 197.00±37.00 | 198.00±21.00 | 178.00±38.00 | **0.030** | 1.000 | 0.097 | **0.046** |
|  | 4 week variation ^a^ | 37.00±69.00 | 28.00±51 | 8.00±56.00 | 0.297 | 0.903 | 0.256 | 0.125 |
|  | 13 week variation ^a^ | 40.00±82.00 | 30.00±62.00 | 5.00±78.00 | 0.559 | 0.534 | 0.778 | 0.256 |
| Urea (mmol/L) | Baseline ^b^ | 5.35±1.19 | 5.72±1.25 | 5.13±1.07 | 0.121 | 0.200 | 0.446 | **0.042** |
|  | Week 4 ^b^ | 4.74±0.86 | 4.98±1.11 | 4.93±0.91 | 0.566 | 0.312 | 0.426 | 0.830 |
|  | Week 13 ^b^ | 4.97±1.04 | 5.55±1.07 | 4.94±0.81 | **0.021** | **0.019** | 0.890 | **0.013** |
|  | 4 week variation ^b^ | -0.59±1.23 | -0.74±1.15 | -0.27±1.08 | 0.251 | 0.595 | 0.270 | 0.104 |
|  | 13 week variation ^b^ | -0.43±1.04 | -0.15±1.03 | -0.36±0.93 | 0.509 | 0.597 | 0.699 | 0.886 |
| Creatinine (μ mol/L) | Baseline ^b^ | 76.33±13.11 | 76.09±16.34 | 74.91±13.50 | 0.912 | 0.946 | 0.688 | 0.739 |
|  | Week 4 ^b^ | 76.58±10.96 | 74.00±14.13 | 75.21±11.37 | 0.694 | 0.395 | 0.652 | 0.688 |
|  | Week 13 ^b^ | 71.91±10.73 | 71.91±14.35 | 70.36±12.94 | 0.851 | 1.000 | 0.624 | 0.624 |
|  | 4 week variation ^a^ | 1.00±5.00 | -1.00±9.00 | 1.00±6.00 | 0.238 | 0.378 | 0.343 | 0.105 |
|  | 13 week variation ^a^ | -4.00±10.00 | -3.00±15.00 | -2.00±11.00 | 0.794 | 0.525 | 0.621 | 0.837 |

^a^Skewness distribution was expressed by the median ± interquartile interval (Me ± IQR). The rank sum test was used for comparisons between groups.

^b^Normal variance was homogeneous and expressed by the mean ± standard deviation (X ± S). Single factor analysis of variance was used for comparisons between groups.

^c^Normal variance was uneven and expressed by the mean ± standard deviation (X ± S). The rank sum test was used for inter-group comparisons.

**Table 8. Participant inflammation risk and adipocytokine index changes before and after intervention**

|  |  | Control group | Intervention group 1 | Intervention group 2 | *P* (total) | *P*(Intervention group 1/ Control group) | *P*(Intervention group 2/ Control group) | *P*(Intervention group 1/ Intervention group 2) |
| --- | --- | --- | --- | --- | --- | --- | --- | --- |
| CRP (mg/L) | Baseline ^a^ | 1.60±3.10 | 1.80±1.80 | 2.30±2.50 | 0.490 | 0.812 | 0.449 | 0.218 |
|  | Week 4 ^a^ | 1.50±1.90 | 1.20±1.60 | 1.80±2.90 | 0.080 | 0.210 | 0.332 | **0.025** |
|  | Week 13 ^a^ | 1.80±2.20 | 1.00±1.80 | 1.50±2.40 | 0.607 | 0.397 | 0.995 | 0.379 |
|  | 4 week variation ^a^ | -0.50±1.00 | -0.30±1.50 | 0.00±1.70 | 0.114 | 0.607 | 0.178 | **0.035** |
|  | 13 week variation ^a^ | -0.10±0.80 | -0.40±1.00 | -0.60±1.40 | 0.160 | 0.215 | 0.066 | 0.449 |
| MPO（ng/ml） | Week 13 ^a^ | 6.98±7.65 | 6.94±5.06 | 6.87±4.53 | 0.997 | 0.976 | 0.936 | 0.960 |
| Ox-LDL（ng/ml） | Week 13 ^a^ | 131.38±79.59 | 127.08±69.00 | 104.75±68.31 | 0.888 | 0.883 | 0.705 | 0.649 |
| LEP（ng/ml） | Week 13 ^a^ | 6.65±3.65 | 5.76±3.51 | 6.46±3.58 | 0.574 | 0.318 | 0.828 | 0.434 |
| ADPNN（μg/ml） | Week 13 ^a^ | 15.06±13.42 | 15.44±14.14 | 16.94±15.95 | 0.857 | 0.916 | 0.601 | 0.676 |
| TNF-α（pg/ml） | Week 13 ^a^ | 53.75±38.80 | 51.12±38.34 | 60.57±45.39 | 0.628 | 0.795 | 0.500 | 0.351 |
| TGF-β1 （ng/ml） | Week 13 ^a^ | 4.29±2.18 | 3.85±1.92 | 3.77±1.54 | 0.489 | 0.349 | 0.268 | 0.862 |
| IL-6（pg/mL） | Week 13 ^a^ | 5.57±5.01 | 3.33±2.40 | 5.02±3.62 | 0.051 | **0.019** | 0.557 | 0.077 |
| GPLD1（ug/mL） | Week 13 ^a^ | 17.22±13.67 | 15.93±12.82 | 16.66±12.69 | 0.922 | 0.689 | 0.863 | 0.820 |
| pro-NT（pmol/L） | Week 13 ^a^ | 7.05±6.21 | 6.21±4.03 | 4.81±4.66 | 0.195 | 0.498 | 0.074 | 0.264 |
| GPC-4（pg/mL） | Week 13 ^a^ | 373.36±327.75 | 321.42±276.11 | 319.00±292.35 | 0.708 | 0.483 | 0.463 | 0.974 |
| LPS（pg/mL） | Week 13 ^a^ | 4.01±3.28 | 3.87±3.19 | 3.48±3.14 | 0.787 | 0.857 | 0.505 | 0.626 |

^a^Represented by the mean ± standard deviation (X ± S). Single factor analysis of variance was used for comparisons between groups.
